# An Active Inference view of cognitive control

**DOI:** 10.3389/fpsyg.2012.00478

**Published:** 2012-11-05

**Authors:** Giovanni Pezzulo

**Affiliations:** ^1^Istituto di Linguistica Computazionale “Antonio Zampolli”, Consiglio Nazionale delle Ricerche, via Giuseppe MoruzziPisa, Italy; ^2^Istituto di Scienze e Tecnologie della Cognizione, Consiglio Nazionale delle Ricerche, via S. Martino della BattagliaRoma, Italy

**A commentary on**

**What ever next? Predictive brains, situated agents, and the future of cognitive science**

by Clark, A. (in press). Behav. Brain Sci.

The Active Inference framework (Friston et al., 2009; Friston, 2010) argues that the brain's generative models continuously produce predictions and goals that guide its action (active inference) and perception (predictive coding) through free energy minimization. In this framework, most studies have focused on the on-line prediction of perceptual events and the control of overt behavior. We propose that the framework can be extended to explain cognitive control.

We assume that architectures of cognitive control are elaborations of the predictive architectures of sensorimotor behavior in early living organisms. As the sensorimotor control system of early organisms evolved (to face increasingly harder individual and social problems), it gradually began predicting increasingly long-term and abstract consequence of behavior and—critically—doing so off-line and without overt behavior. This permitted rehearsing action sequences without executing them. In turn, off-line predictions opened the doors to higher cognitive abilities, such as planning, emulation, imagery, mental state inference, goal-directed decision-making, prospection, and the acquisition of declarative knowledge (Hesslow, 2002; Grush, 2004; Jeannerod, 2006; Pezzulo and Castelfranchi, 2007, 2009; Moulton and Kosslyn, 2009; Pezzulo, 2011; Clark, in press).

We argue that off-line predictions opened the doors to executive functions and cognitive control, too. Executive functions, typically linked to prefrontal cortex, are self-directed actions functional to self-regulation and the coordinated planning of present and future actions and goals toward distal objectives. Executive functions (and prefrontal cortex functioning) have been linked to a plethora of processes, including the self-generation of goals and plans, their maintenance in working memory, their monitoring, the inhibition of prepotent but inappropriate actions, the regulation of attention, and valuation processes. A reconciling perspective is that prefrontal cortex supports “cognitive control”: the control of thought processes and the top–down guidance of overt behavior toward distal goals (Fuster, 1997; Miller and Cohen, 2001; Botvinick, 2008).

**Figure 1:**
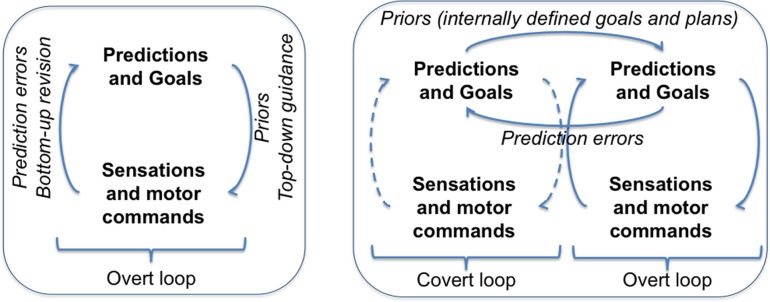
**Left:** Active Inference framework. **Right:** “Controlled Predictions” and cognitive control.

We cast cognitive control within the Active Inference framework. We argue that cognitive control consists in a *nesting* of optimizations (i.e., free energy minimization loops) over time; in addition to the usual overt loop of active inference, one (or more) covert loop(s) help optimizing distal goals. The left part of Figure 1 shows the Active Inference framework, in which predictions and goals steer perception and action via free energy minimization. Note that here predictions are relative to the present situation. The right part of Figure 1 shows an extension of the framework that includes Controlled Predictions and a nesting of optimizations (for simplicity we only show two loops).

The covert loop works off-line via the suppression of overt sensory and motor processes (in the Active Inference framework, this requires the suppression of proprioception). This permits running imaginary actions that produce a sequence of fictive actions and of predictions relative to future (rather than present) situations. Fictive actions and predictions can be optimized via free energy minimization but without overt execution: they are not just “mind wandering” but are truly *controlled* toward goals specified at higher hierarchical levels. Prospection and planning are thus optimization processes that support the generation of distal and abstract goals (and associated plans), beyond current affordances. The covert loop supports also the recall of learned contextual goals and rules rather than always forming them *de novo* via active inference.

The selected goals can thus be set as *priors* for the overt loop. In the Active Inference framework, this has the same effect as goal maintenance in working memory, and affords the top–down guidance of overt behavior beyond stereotyped responses, which is the hallmark of cognitive control. [Note that specifying contextual rules or plans to distal goals requires setting strong priors over sets of states and transitions rather than just one set point (Friston et al., 2012)].

Active inference in the overt loop ensures that the goals (and plans) generated by the covert loop are achieved in practice. In turn, feedback from the overt loop is informative of (changing) environmental constraints; it permits revising and re-situating imagined goals, and ultimately achieving them in the current context. In some cases, the overt loop is recruited for planning and thinking, too, such as for instance when Tetris players rotate blocks to better decide where to place them (Kirsh and Maglio, 1994).

Our view is compatible with theories of cognitive control that highlight the active maintenance of goals in working memory (Miller and Cohen, 2001). However, we assume that cognitive control and mental operations are internalized forms of overt actions and recruit the same brain structures, rather than involving separated neural representations and dedicated, modular processing (Diamond, 2000; Barkley, 2001; Cotterill, 2001; Ito, 2008; Pezzulo, 2011).

We propose that the nesting of optimization processes is mainly realized in prefrontal hierarchies and is functional to the achievement of goals at different time scales. Compared to overt action, cognitive control optimizes at longer time scales, and requires maintaining goal representation (priors) for a long time. It is thus not surprising that cognitive control processes (and in our proposal, covert loops) mainly involve high hierarchical levels. This creates a potential confound, suggesting that cognitive control might also require dedicated neural resources or modularized processing. However, our proposed architecture can be implemented within a homogeneous (cortical) hierarchy performing free energy minimization; which ones of the hierarchical levels are recruited to work covertly depends on task demands (say, abstract thought vs. the mental imagery of rotating an object with the left hand).

Our view suggests that executive functions and forethought might have resulted from an internalization of the process of predicting the consequences of actions, which permitted to endogenously steer and control covert predictive loops. Partial support for this view comes from the close relationships between the neural systems for motor preparation and mental imagery (Cisek and Kalaska, 2010). Furthermore, in our proposal cognitive control requires the coordination of overt and covert processes. Disruption of this process might result in the execution of imagined actions, as it was reported by Schwoebel et al. (2002) in the case of a patient with bilateral parietal lesions who was unable to refrain from executing imagined (hand) movements.
